# Distinctive collagen maturation process in fibroblasts derived from rabbit anterior cruciate ligament, medial collateral ligament, and patellar tendon in vitro

**DOI:** 10.1007/s00167-013-2773-8

**Published:** 2013-11-13

**Authors:** Soki Kato, Mitsuru Saito, Hiroki Funasaki, Keishi Marumo

**Affiliations:** Department of Orthopaedic Surgery, The Jikei University School of Medicine, 3-25-8 Nishi-shinbashi, Minato-ku, Tokyo 105-8461 Japan

**Keywords:** Cell culture, Collagen, Cross-linking, Fibroblast, Ligament, Tendon

## Abstract

**Purpose:**

Differences in the tissue-specific collagen maturation process between tendon and ligament are still unknown. Collagen cross-link formation is crucial for the collagen maturation process. The aim of this study is to examine collagen maturation processes of anterior cruciate ligament (ACL), medial collateral ligament (MCL), and patellar tendon (PT) in vitro, in order to determine the optimal cell source for tissue engineering of ligament.

**Methods:**

Cells derived from the ACL, MCL, and PT of New Zealand white rabbits were isolated. Each cell type was cultured for up to 4 weeks after reaching confluence. Cell–matrix layers were evaluated and compared for their morphology, collagen cross-links, and gene expression levels of lysine hydroxylase 1 and 2, lysyl oxidase (LOX), tenomodulin, collagen1A1 (Col1A1), and collagen3A1 (Col3A1).

**Results:**

Transmission electron microscopy photomicrographs verified that collagen fibrils were secreted from all three types of fibroblasts. A higher ratio of dihydroxylysinonorleucine/hydroxylysinonorleucine was evident in the ligament compared to the tendon, which was consistent with lysine hydroxylase 2/lysine hydroxylase 1 gene expression. The gene expression of LOX, which regulates the total amount of enzymatic cross-linking, and the gene expression levels of Col1A1 and Col3A1 were higher in the ACL matrix than in the MCL and PT matrices.

**Conclusion:**

ACL, MCL, and PT cells have distinct collagen maturation processes at the cellular level. In addition, the collagen maturation of ACL cells is not necessarily inferior to that of MCL and PT cells in that all three cell types have a good ability to synthesize collagen and induce collagen maturation. This bioactivity of ACL cells in terms of ligament-specific mature collagen induction can be applied to tissue-engineered ACL reconstruction or remnant preserving procedure with ACL reconstruction.

## Introduction

The anterior cruciate ligament (ACL) is one of the most frequently injured structures in the knee. Disruption of the ACL leads to functional instability, meniscal injury, and articular surface damage. Some studies have reported that ACL-derived fibroblasts are inferior to medial collateral ligament (MCL)- and patellar tendon (PT)-derived fibroblasts in terms of their proliferation [4, 6, 20, 29, 41], migration [9, 16, 23, 29, 41], and collagen synthesis [7, 20, 41]. Moreover, ACL cells are thought to have relatively poor healing capabilities. This poor healing capacity leads to the need for ACL reconstruction in many cases, and more than 100,000 ACL reconstructions are performed every year in the USA [21]. Autologous tendon grafts (the bone-patellar tendon-bone graft, the hamstring tendons, and the quadriceps tendon) are most commonly used in ACL reconstruction because tendons and ligaments are grossly similar, both being dense, white, and composed mainly of type I collagen.

Despite their gross similarities, tendons and ligaments have unique histological and biochemical characteristics [1, 14, 19, 33]. The histological and ultrastructural appearance of the human ACL differs from that of tendons used as grafts for ACL reconstruction (gracilis, semitendinosus, quadriceps, and PT) [19]. It was previously reported that tissue-specific collagen cross-link formation, a distinctive biological feature of collagen fibres, occurs in ligaments and periarticular tendons of the human knee [14, 27, 34]. Collagen cross-link formation directly affects the strength of bone, tendon, and ligament [13, 34, 40]. On the basis of human biopsy data, 1 year after ACL reconstruction, the collagen cross-link patterns of the tendon autografts were very similar to those of native ACL [27]. Recently, tenomodulin has been used as an indicator for ligamentogenic differentiation of mesenchymal progenitor cells [18], although it is expressed in both tendons and ligaments [22]. Collagen cross-link analysis enabled us to determine whether collagenous tissue differentiated into ligament or tendon. The distinct collagen cross-linking pattern is regulated by lysine hydroxylase (procollagen-lysine, 2-oxoglutarate 5-dioxygenase; PLOD) and lysyl oxidase (LOX), which are both secreted by tissue cells. These enzymes perform specific functions depending on the demands of the body [34].

Moreover, the primary concern about autologous tendon grafts is the donor site morbidity. The use of allografts or permanent synthetic ACL prostheses can be used to overcome this. However, allografts are associated with risks of inflammatory reaction and infection [25]. Permanent synthetic ACL prostheses have poor long-term results [5, 17, 28, 31]. Various studies in ligament tissue engineering have recently been undertaken to resolve these disadvantages [8, 15, 26, 39]. Selection of an appropriate cell source for ligament tissue engineering is one of the primary goals of research, besides material selection for tissue engineering scaffolds. The cell type used for ACL tissue engineering must show enhanced proliferation and production of appropriate extracellular matrix, and must be able to survive in an intraarticular environment in the patient’s knee. However, little is known about the quality of collagen produced, resulting from cell source selection.

It is important to understand the distinct intrinsic cellular and tissue properties of ligaments and tendons when selecting an appropriate cell source for tissue-engineered ACL reconstruction. The first purpose of this study was to determine the optimal cell source for tissue engineering of ligament with regard to the cross-links of the matrices, the related gene expressions of LOX, PLOD, and the gene expression of matrix markers such as Collagen1A1 (Col1A1) and Collagen3A1 (Col3A1). The second purpose was to confirm the tissue-specific collagen maturation process at the cellular level with regard to cross-link formation of the matrices and cellular morphology with time, and the gene expression of tenomodulin. To address these issues, the collagen maturation process in fibroblasts derived from rabbit ACL, MCL, and PT in vitro was investigated in this study. The hypotheses were as follows. First, ACL-derived cells form lower quality collagenous matrices than MCL- and PT-derived cells and are not adequate for ACL tissue engineering as the cell source. Second, ACL, MCL, and PT-derived cells have distinct collagen maturation processes at the cellular level.

## Materials and methods

### Harvesting of tissues and cells

Seven skeletally mature 12-week-old female New Zealand white rabbits (weighing 2.5 ± 0.1 kg) were killed by an intravenous bolus injection of sodium pentobarbital (more than 100 mg/kg). The knee was exposed by a medial parapatellar incision. The ACL, MCL, and PT were harvested under aseptic conditions and collected into sterile tissue culture medium. All further steps were conducted in a sterile biological safety hood. The synovial sheath was carefully scraped off with a sharp blade. The remaining tissue was scraped again, and the origins and insertions were removed to ensure that a homogeneous preparation of ligament and tendon mid-substance was available for enzymatic digestion [32]. The ligaments and tendons were then transferred to a new dish and were diced into pieces approximately 1–2 mm^3^ using no. 15 scalpels. The tissue was digested with 0.1 % collagenase (no. C-0130, Sigma, St.Louis, MO) prepared in Dulbecco’s modified Eagle’s medium (DMEM, Gibco, Life Technologies Corp., Carlsbad, CA, USA) without any supplements for 20 h in a water bath maintained at 37 °C.

### Cell culture

After digestion, the cells were washed twice with culture medium, centrifuged at 200×g, then resuspended in DMEM (Gibco) + 10 % foetal bovine serum (FBS, Thermo Fisher Scientific, Logan, UT, USA) + 100 IU/ml penicillin and 100 μg/ml streptomycin (Gibco, Life Technologies Corp., Carlsbad, CA, USA) + 50 μg/ml l-ascorbic acid (no. 013–12061, Wako) [40]. The suspension was strained through a 100 μm cell strainer (BD Biosciences, Billerica, MA, USA) into a 100 mm tissue culture dish (Iwaki Co. Ltd, Tokyo, Japan), which was then transferred to a humidified incubator at 37 °C with a 5 % CO_2_ atmosphere. The cell culture medium was replaced twice a week. Cells were passaged into a 100 mm tissue culture dish for collagen cross-link analysis and 35 mm tissue culture dishes for DNA, RNA, and transmission electron microscopy (TEM) analyses.

Cells obtained at passage 2 were used in this study. After reaching confluence, the cells were cultured for up to 4 weeks. The DNA assay was performed at confluence (*n* = 5). Real-time reverse transcription polymerase chain reaction (real-time RT-PCR) was used to examine the cellular gene expression levels after 3 weeks of culture (*n* = 5). Collagen cross-link analysis was performed in native tissues (*n* = 7) and in matrices formed by ACL-, MCL-, and PT-derived cells after 1, 2, and 4 weeks of culture (*n* = 5). The cellular morphology characterization was accomplished using TEM at 0, 2 and 4 weeks of culture (*n* = 2).

### Double-stranded DNA assay

Confluency was defined as the condition when a culture dish was completely covered with cells. It was difficult to count the number of cells because they aggregated and contained many proteins. When the cells reached confluence, the double-stranded DNA content was measured using Hoechst33258 instead of counting the number of cells per dish [24]. In brief, the cells and the matrices were collected in a 1.5 ml tube and then homogenized in 10 mM Tris–HCl (pH 7.4). The DNA content of the homogenate was determined using a commercially available cell proliferation DNA assay, the Fluorescent DNA Quantitation Kit (Bio-Rad Laboratories, Inc., Hercules, CA). Fluorescence was measured using a spectrofluorometer (FP-6200, Jasco, Tokyo, Japan), whose excitation wavelength was set at 360 nm and emission wavelength at 460 nm (*n* = 5).

### Collagen cross-link analysis

The other tissue pieces and matrices were used for quantitative analysis of immature divalent and mature trivalent collagen cross-links by high-performance liquid chromatography (HPLC) using a fluorescence detection method established previously in our laboratory [35]. Sample preparation for HPLC was as follows: All tissues were minced and suspended in 500 vol (v/wt) of 0.05 M potassium phosphate buffer, pH 7.6 (ionic strength = 0.15 M) at 4 °C and continuously stirred for 72 h under a vacuum. A 1/30 volume of sodium borohydride (NaBH_4_) was added to the solution. The reaction was allowed to proceed for 60 min at 37 °C and was terminated by the addition of 3 N acetic acid to decrease the pH value to 4.0. The solution residue was collected by centrifugation (3,000g, 15 min), washed with deionized water, and lyophilized. For HPLC analysis, lyophilized samples were dissolved in 0.2 N sodium citrate buffer (pH 2.20) and filtered through a 0.45-μm filter (Gelman Science Japan Ltd, Tokyo, Japan). The analyses of enzymatic divalent immature cross-links, such as dihydroxylysinonorleucine (DHLNL) and hydroxyllysinonorleucine (HLNL), and mature pyridinium cross-links, such as pyridinoline (PYD) and deoxypyridinoline (DPD) and non-enzymatic cross-linking pentosidine (Pen), were performed on a single-column HPLC. The ratio of DHLNL/HLNL was assessed as a surrogate marker of degree of hydroxylation of lysine in collagen molecules. This ratio is distinctively regulated in various collagenous tissues [34].

### Development of rabbit primers for real-time RT-PCR

Total RNA was prepared from cultured rabbit MCL or PT cells with the SV Total RNA Isolation System (Promega, Madison, WI, USA). First-strand cDNA was synthesized using an Oligo(dT)_15_ Primer (Promega) and SuperScript™ III Reverse Transcriptase (Invitrogen, Life Technologies Corp., Carlsbad, CA, USA). We cloned the rabbit LOX, PLOD1, PLOD2 and tenomodulin cDNA fragments on the basis of the human, rat, and mouse cDNA sequences. The resulting amplicons were sequenced (Sigma DNA sequencing service). A National Center for Biotechnology Information (NCBI) search with the Basic Local Alignment and Search Tool (BLAST) confirmed the identity of the genes in question. TaqMan MGB gene expression detection kits (a probe and 2 primers) were designed using these sequences (LOX, PLOD1, PLOD2 and tenomodulin) and consensus rabbit sequences for Col1A1, Col3A1, and glyceraldehyde-3-phosphate dehydrogenase (GAPDH) found in Genbank (Table 1).

**Table 1:** Development of rabbit primers for real-time RT-PCR

Gene	Primer sequences	
Col1A1	FAM-MGB	CCATCAAGGTCTTCTG
	Forward	CCAAGGCTGCAACCTGGAT
	Reverse	TTGCCCCAGTGTCCATGTC
Col3A1	FAM-MGB	TGTGTTCCTTTTGTTCTAAT
	Forward	CCAGCAGAAAATTGCACATTTTAT
	Reverse	AGTTGGTCACTTGTACTGGTTGACA
LOX	FAM-MGB	TTAGCGTCAACCCC
	Forward	CCCGGCAACTACATTCTGAAG
	Reverse	CGACTCGGGCACCAGGTA
PLOD1	FAM-MGB	CCAACCATCGACATCC
	Forward	TCCAGGGTGGGTATGAGAATGT
	Reverse	GCTCGAAGCTGATCTGGTTCA
PLOD2	FAM-MGB	CTTCTACCTTTACCATCAAC
	Forward	TCTCTCCGTCCTCACCATGAC
	Reverse	TCTCCCACGTTATTGAGTGCAA
Tenomodulin	FAM-MGB	CCCTGACTCTAATTGTC
	Forward	GTGGACTGGTGTTCGGTATCCT
	Reverse	GGCCAGAAGTACTTACTACCCCAAA
GAPDH	FAM-MGB	CACATGGCCTCCAAGG
	Forward	GGGTGGTGGACCTCATGGT
	Reverse	CGGTGGTTTGAGGGCTCTTA

Col1A1 (AY633663), Col3A1 (S83371), and GAPDH (L23961) from GenBank

### Quantitative real-time RT-PCR

RNA was isolated and used to characterize the gene copy number within the cells by RT-PCR. Total RNA was isolated using the RNeasy Fibrous Tissue Mini Kit (Qiagen Inc., Hilden, Germany), and first-strand cDNA was synthesized using a high-capacity cDNA Reverse Transcription Kit (Applied Biosystems, Life Technologies Corp., Carlsbad, CA, USA). Real-time PCR was performed in triplicate using the TaqMan Gene Expression Master Mix according to the manufacturer’s specifications (Applied Biosystems) and specific primer sets for Col1A1, Col3A1, LOX, PLOD1, PLOD2, tenomodulin, and GAPDH. Samples were analysed by the Applied Biosystems StepOne™ Real-Time PCR System (Applied Biosystems). The housekeeping gene, GAPDH, which is present in all cells, was used as an internal control to normalize the data because it has an unchanged or constant level of expression in all samples. The transcription level of target genes normalized to GAPDH was then calculated using the 2^Δ^ Ct formula with reference to the ACL-, MCL-, and PT-derived cells.

### Cellular morphology

Characterization of cellular morphology was accomplished using TEM (time points: 0, 2, and 4 weeks) (*n* = 2). Cells were doubly fixed with 2 % glutaraldehyde/0.1 M phosphate buffer (PB) (pH 7.4) and 1 % osmium tetroxide/0.1 M PB (pH 7.4) and were dehydrated through a graded series of ethanol. Cells were then embedded in epoxy resin. Ultrathin sections were stained with uranyl acetate and lead citrate and were observed with a Hitachi H-7500 transmission electron microscope.

All experimental procedures were approved by the animal care committee of the Jikei University School of Medicine. The ID number of the approval was 20-041.

### Statistical analysis

A one-factor analysis of variance (ANOVA) was performed to compare the fibroblast types (ACL, MCL, and PT) or tissue types (ACL, MCL, and PT), followed by a Tukey’s post hoc test to determine the significance for the DNA content per well at confluence, cross-links of collagen at 1, 2, and 4 weeks after reaching confluence, and the relative abundance of target genes at 3 weeks after reaching confluence. Statistical significance was established at *p* < 0.05. The test–retest reliability was determined using the coefficient of variation, which expresses the extent of variability between the measurements. The coefficient of variation in HPLC and real-time PCR is approximately 1 and <1 %, respectively, based on the data obtained in preliminary experiments [35].

## Results

### DNA content

**Fig. 1:**
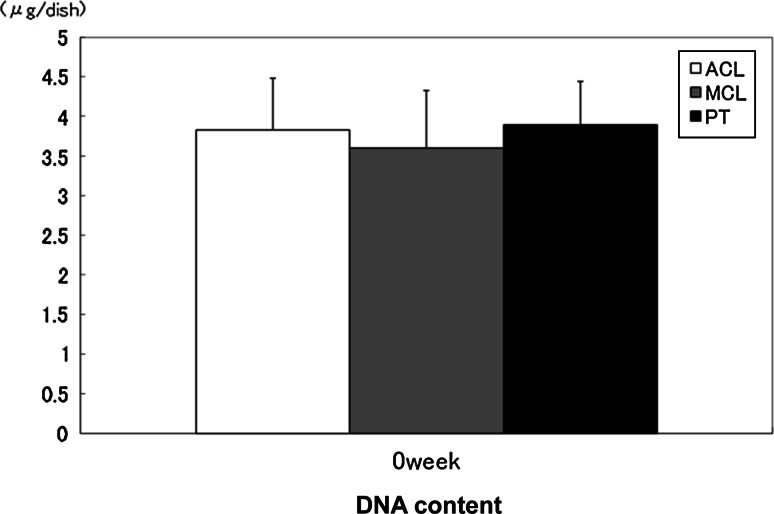
DNA content was determined using Hoechst 33258 staining to compare cell counts between ACL, MCL, and PT cells at confluence. The *error bars* represent the standard deviation of the mean of five samples harvested from five separate populations. There was no significant difference in the cell count at confluence between these cell types

There were no significant differences in the DNA content between the ACL, MCL, and PT matrices when the cells reached confluence (Fig. 1).

### Collagen cross-linking

**Fig. 2:**
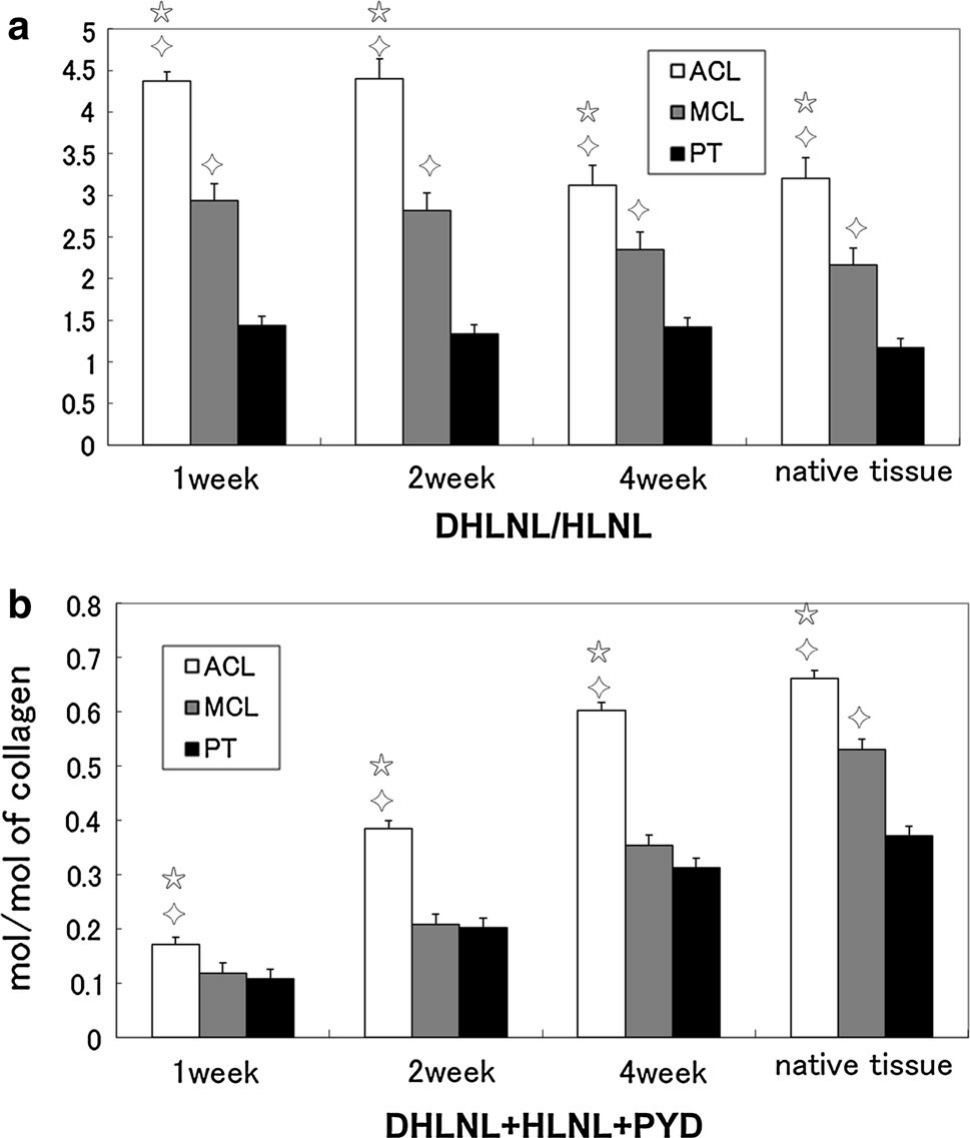
Collagen cross-links were examined by high-performance liquid chromatography (HPLC). **a** The DHLNL/HLNL ratio in the matrices formed by ACL- and MCL-derived cells was significantly higher than in the matrix formed with PT-derived cells. **b** The total amount of enzymatic cross-linking (DHLNL + HLNL + PYD) of collagen was significantly higher in the ACL matrix than in the MCL and PT matrices. These results were consistent with those of native tissues. (*star* significant difference compared to MCL-derived cells and *diamond* significant difference compared to PT-derived cells (*p* < 0.05). The *error bars* represent the standard deviation of the mean of five samples harvested from five separate populations at 1, 2, and 4 weeks after reaching confluence in culture, and seven samples harvested from seven separate native tissues

The DHLNL/HLNL ratio, which is an indicator of tissue-specific collagen maturation, in the matrices formed by ACL- and MCL-derived cells was significantly higher than in the matrix formed by PT-derived cells (all *p* < 0.05) (Fig. 2a). The ratio of DHLNL/HLNL of native tissues was also confirmed. The DHLNL/HLNL ratio in the native rabbit ACL and MCL was significantly higher than in the native rabbit PT (all *p* < 0.05) (Fig. 2a). The DHLNL/HLNL ratio of rabbit ligament was significantly higher than rabbit tendon, which is in agreement with a previous human study [27]. PYD was detected in the ACL, MCL, and PT matrices, but neither DPD nor Pen was detected in these matrices. The total amount of enzymatic cross-linking (the sum of DHLNL, HLNL, and PYD) of collagen was significantly higher (*p* < 0.05) in the ACL matrix than in the MCL and PT matrices (all *p* < 0.05). The total amount of enzymatic cross-linking of the ACL, MCL, and PT matrices increased time-dependently (Fig. 2b). The total amount of enzymatic cross-linking of the ACL and PT matrices at 4 weeks was similar to native tissues (Fig. 2b).

### Gene expression

Gene expression levels were examined to clarify the regulation of collagen cross-link formation. We investigated gene expression at 3 weeks of culture because TEM photomicrography at 4 weeks showed destruction of cell membranes. This was thought to be the time limit for culturing these cells in a culture dish.

**Fig. 3:**
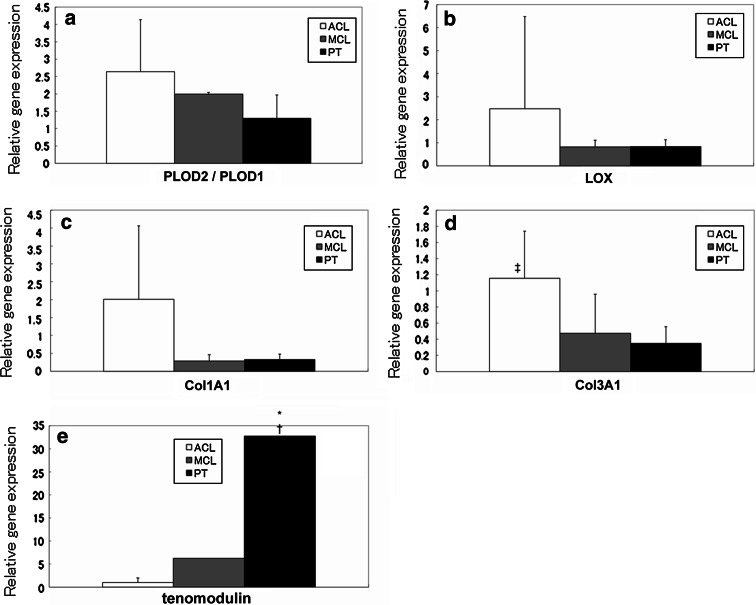
Gene expression levels of PLOD2/PLOD1 (**a**), LOX (**b**), Col1A1 (**c**), Col3A1 (**d**), and tenomodulin (**e**) were determined by real-time reverse transcription polymerase chain reaction (real-time RT-PCR). The expression levels were relative to that of ACL-derived cells. The *error bars* represent the standard deviation of the mean in cells harvested in triplicate from five separate populations 3 weeks after reaching confluence in culture. Samples were normalized to glyceraldehyde-3-phosphate dehydrogenase (GAPDH) levels for each cell type. The gene expression levels of PLOD2/PLOD1 (**a**) and LOX (**b**) were consistent with the results of the cross-linking patterns and the total amount of enzymatic cross-linking, respectively. The gene expression levels of Col1A1 (**c**) and Col3A1 (**d**) were higher in the ACL matrix than in the MCL and PT matrices. The gene expression level of tenomodulin (**e**) was significantly higher in the PT matrix than in the ACL and MCL matrices. [*significant difference compared to ACL, ^†^significant difference compared to MCL and ^‡^significant difference compared to PT (*p* < 0.05)]

The PLOD2/PLOD1 ratio, which regulates distinct collagen cross-linking patterns, was significantly higher in the ACL and MCL matrices than in the PT matrix (n.s. and *p* < 0.05, respectively) (Fig. 3a). The gene expression of LOX and Col1A1 showed higher trend in the ACL matrix than in the MCL and PT matrices (Fig. 3b, c). The expression of the Col3A1 gene was significantly higher in the ACL matrix than in the MCL and PT matrices (n.s. and *p* < 0.05, respectively) (Fig. 3d). The expression of the tenomodulin gene was significantly higher in the PT matrix than in the ACL and MCL matrices (both *p* < 0.05) (Fig. 3e).

### Cellular morphology

**Fig. 4:**
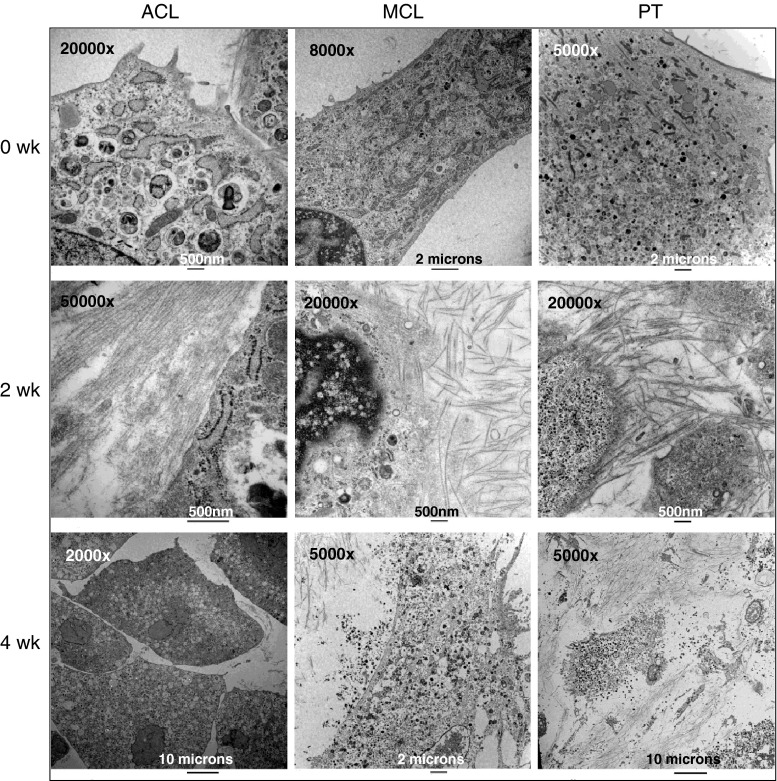
Transmission electron microscopy (TEM) photomicrographs showed collagen fibrils secreted from rabbit anterior cruciate ligament (ACL), medial collateral ligament (MCL), and patellar tendon (PT) cells at 0, 2, and 4 weeks after reaching confluence in culture. The TEM photomicrographs showed a random orientation of the deposited fibrous matrix. The cytoplasm of the cells contained large quantities of rough-surfaced endoplasmic reticulum when the cells reached confluence in culture. Two weeks after reaching confluence, all of the cells secreted large amounts of collagen. However, the cytoplasm of ACL cells contained many lacunae. After 4 weeks in culture, the plasma membrane of the MCL and PT cells had ruptured releasing the contents of the cytoplasm from the cells because of deterioration in the conditions of the culture environment

TEM photomicrographs showed collagen fibrils secreted from rabbit ACL, MCL, and PT cells at 0, 2, and 4 weeks after reaching confluence in culture. TEM photomicrographs showed a random orientation of the deposited fibrous matrix. The cytoplasm of the cells contained large quantities of rough-surfaced endoplasmic reticulum when the cells reached confluence. Rough-surfaced endoplasmic reticula synthesize proteins. After 2 weeks in culture, all three types of fibroblasts secreted a large amount of collagen. However, the cytoplasm of ACL cells contained many lacunae. After 4 weeks in culture, the plasma membrane of the MCL and PT cells had ruptured, releasing the contents of the cytoplasm from the cells because of deterioration in the conditions of the culture environment (Fig. 4).

## Discussion

The most important finding of the present study was that ACL, MCL, and PT cells have distinct collagen maturation processes at the cellular level and that collagen maturation of ACL cells is not necessarily inferior to that of MCL and PT cells. Collagen cross-link formation, which is crucial for the tissue-specific collagen maturation process, may occur distinctively in ligaments and periarticular tendons of the knee even at cellular level. Therefore, in this study, the collagen cross-links in the extracellular matrices secreted by rabbit ACL, MCL, and PT cells were examined to evaluate whether the distinctive matrix maturation process occurs at both the cellular and tissue levels. These findings are important information not only for understanding the tissue maturation process, but also in selecting a cell source for tissue-engineered ACL replacement.

Previous studies demonstrated that ACL proliferation and migration rates were slower than MCL or PT fibroblasts under various conditions [4, 6, 7, 9, 16, 20, 23, 29, 41]. In this study, rabbit fibroblasts (ACL, MCL, and PT) were cultured in a culture dish for up to 4 weeks after reaching confluence in order to eliminate differences between the cell types. When the cells reached confluence, double-stranded DNA content was measured instead of cell counts. No difference was noted in the DNA content between the ACL, MCL, and PT matrices. Thus, each cell surrounded with a mature collagenous matrix may display similar growth characteristics in long-term cultures.

Some previous studies have shown that there are morphological differences between tendons and ligaments both in vivo [1, 19, 33] and in vitro [4, 7]. Tendons and ligaments have a characteristic wavy pattern, the periodicity and amplitude of which is greater in tendon in vivo [1]. Tendons are hypocellular in comparison with ligaments and contain thinner, spindle-shaped fibroblasts [1]. ACL-derived cells are more ovoid in shape and larger in diameter, while MCL-derived cells adopt a spindle-shaped form in vitro [4, 7]. However, we found no significant morphological differences between ACL-, MCL-, and PT-derived cells and matrices in vitro. They all appeared as spindle-shaped forms. None of the collagen fibrils secreted from any of the three types of cells had a characteristic wavy pattern. The discrepancies between this study and the previous studies may be explained by differences in culture conditions and period of incubation. Chun et al. showed that fibroblasts (ACL or MCL) incubated in 10 % FBS or TGF-β1 were more spindle-shaped compared to fibroblasts incubated in 0.5 % FBS. The differences in morphology were most noticeable when they were incubated in 0.5 % FBS [7]. In terms of morphological and biochemical maturity, our study shows that ACL cells are candidates for tissue-engineered ACL replacement as they have the ability to produce mature ligamentous collagen fibres.

Rabbit ligaments and tendons have differences in collagen maturation according to the age of the rabbit [3]. Twelve-week-old NZW rabbits were used in this study because they were used in previous ex vivo studies [10–12]. DPD is present in mature pyridinium cross-links and is relatively indicative of calcified tissue. Pen is an advanced glycation end product, and it accumulates with ageing [34]. Neither DPD nor Pen was detected in the matrices formed by ACL-, MCL-, and PT-derived cells, or in native tissues (12-week-old rabbit ACL, MCL, and PT). A higher ratio of DHLNL/HLNL in ligaments, regulated by PLOD2 and PLOD1 gene expressions, was observed compared to tendon in vitro, which was in agreement with a previously reported in vivo study [1, 2, 14, 34]. Differences in the tissue-specific ratio of DHLNL/HLNL in the matrices were evident at 1 week after reaching confluence. These results suggest that ACL, MCL, and PT cells have a distinct collagen maturation process at the cellular level.

It is generally accepted that the total amount of enzymatic cross-linking is controlled by the expression of LOX. There have been several previous studies showing that collagen cross-link formation directly affects the strength of bones, tendons, and ligaments [34]. The total amount of enzymatic cross-linking in a tendon or ligament is the sum of enzymatic divalent immature cross-links (DHLNL and HLNL) and mature pyridinium cross-links (PYD). In this study, the total amount of cross-linking is represented as the number per collagen molecule. The total amount of enzymatic cross-linking was higher in the ACL matrix than in the MCL and PT matrices both in vivo and in vitro, which was consistent with LOX gene expression. Furthermore, Col1A1 and Col3A1 mRNA expression was detected in all fibroblast types as typical markers of differentiation. However, ACL fibroblasts had greater up-regulation of Col1A1 and Col3A1 gene transcription compared to the other fibroblast types. Cooper et al. [8] suggested that ACL cells are the most suitable cell type for ligament tissue engineering as they have the highest gene expression of matrix markers (collagen I, collagen III, and fibronectin). We demonstrated that collagen gene expression increased in ACL fibroblasts and that expression of genes involved in regulating collagen cross-links was higher than in other ligament and tendon cell types.

In this study, the gene expression levels of the non-collagenous protein tenomodulin were examined. Tenomodulin is a member of a recently identified family of type II transmembrane glycoproteins. It is predominantly expressed in tendons, ligaments, and eyes [22]. The C-terminal domain of tenomodulin exhibits anti-angiogenic activity when it is expressed in a secreted form. Tenomodulin transcripts have been found in hypovascular tissues such as tendons and ligaments [30]. Ligaments and tendons are similar in composition but differ histologically. Hadjicostas et al. [19] showed that the density of blood vessels in the human ACL is higher than in human semitendinosus gracilis, patellar, and quadriceps tendons. This study also showed that tenomodulin expression in cultured PT cells was significantly higher than in cultured ACL and MCL cells. This indicates that the rabbit PT may also be more hypovascular than the rabbit ACL and MCL in vivo. Tenomodulin has recently been used as an indicator of ligamentogenic differentiation of mesenchymal progenitor cells [18]. However, since tenomodulin is expressed in both tendon and ligaments, it cannot be used as a specific discriminating marker to determine whether the matrix differentiates into a tendon or ligament. In contrast, the combination of the DHLNL/HLNL ratio and the presence/absence of DPD cross-links may enable us to discriminate between ligament, tendon, and calcified tissue.

There are limitations that must be addressed when interpreting the data. First, the reported results may be species-specific, and therefore, the findings may not be applicable to the clinical setting. However, the rabbit model has been widely utilized in previous studies. In addition, the results from these rabbit studies were similar to previous human studies. For the present study, only mRNA levels were assessed and it is unclear if protein amounts were also altered. However, we have previously reported that gene expression levels of LOX and PLOD correlated with the total amount of enzymatic cross-linking (DHLNL + HLNL + PYD) and cross-linking patterns [36, 37]. Such findings support the contention that an increase in mRNA levels, as was found in this study, is indicative of increased amounts of functional protein. However, confirmation at the protein level would unequivocally define the roles of these molecules and their function in the collagen maturation process. The collagen fibrils secreted by these fibroblasts were non-oriented because the cells were cultured under conditions without dynamic stress. Therefore, more studies are necessary to investigate not only collagen morphology, but also the cross-links of collagen in three-dimensional cultures on a scaffold and/or using a bioreactor. The sample size of 7 was selected as previously reported studies have utilized 4–6 samples. However, for greater accuracy, a larger sample is warranted.

The present findings permit two clinical applications. First, future studies should focus on using ACL cells as a source for tissue-engineered ACL replacement. ACL cells abundant in the injured ACL can be easily harvested from the patient arthroscopically, and at the same time, the meniscus can be repaired if injured. After the tissue engineering ACL is generated from the ACL-derived cells, a second arthroscopic surgery for ACL reconstruction can be performed.

Second, a remnant preserving procedure with ACL reconstruction using a tendon graft may result in ACL type collagen formation from the graft. The procedure has been attempted for some advantages such as healing potential, preserved proprioception, and revascularization [38]. It is possible that future exploitation of the present study will lead to important progress in ligament tissue engineering.

## Conclusion

This study demonstrates that ACL, MCL, and PT-derived cells have distinct collagen maturation processes at the cellular level and that collagen maturation of ACL cells is not necessarily inferior to that of MCL and PT cells with regard to collagen synthesis and maturation.
